# The Rac1-inhibitor EHop-016 attenuates AML cell migration and enhances the efficacy of daunorubicin in MOLM-13 transplanted zebrafish larvae

**DOI:** 10.1016/j.tranon.2024.101876

**Published:** 2024-01-06

**Authors:** Anette Lodvir Hemsing, Jan-Lukas Førde, Håkon Reikvam, Lars Herfindal

**Affiliations:** a Department of Medicine, Haukeland University Hospital, pb 1400, Bergen 5021, Norway; b Department of Clinical Science, University of Bergen, Jonas Lies vei 87, Bergen 5021, Norway; c Centre for Pharmacy, Department of Clinical Science, University of Bergen, Jonas Lies vei 87, Bergen 5021, Norway

**Keywords:** AML, Rac1, Zebrafish larvae, Cell migration, Homing, Therapy

## Abstract

Ras-related C3 botulinum toxin substrate 1 (Rac1) is a GTPase implicated in cell migration and homing of hematopoietic cells to the hematopoietic niche, and is commonly overexpressed in acute myeloid leukemia (AML). This can lead to quiescence of leukemic blasts in the niche and reduced response to therapy. We investigated the Rac1 inhibitor EHop-016 on AML by assessing its effects on MOLM-13 cells *in vitro* and in zebrafish larvae, regarding cell motility and therapeutic potential in combination with daunorubicin (DNR). *In vitro* assessment of proliferation and viability was by measurement of ^3^H-thymidine incorporation and detection of Annexin V/PI positive cells. Cell motility was evaluated by measurement of migration in a transwell system. Fluorescently stained MOLM-13 cells were injected into zebrafish larvae, and individual cells followed by confocal microscopy. Cell accumulation in the caudal hematopoietic tissue (CHT) was studied using a 12-hour timelapse, while *in vivo* efficacy of DNR, EHop-016 or a combination was investigated over 24 h.

The *in vitro* results showed that EHop-016 acted synergistically in combination with DNR in reducing the viability of MOLM-13 cells (Bliss synergy score above 10 %). Non-toxic concentrations of EHop-016 reduced cell migration. These findings were reproduced in zebrafish larvae: larvae receiving both DNR and EHop-016 had significantly reduced tumor burden compared to the untreated control or single treatments. The accumulation of MOLM-13 cells in the CHT was reduced in larvae receiving EHop-016 treatment.

Our findings demonstrate that targeting Rac1 in AML holds promise as a complementary treatment to established chemotherapy and should be further investigated.

## Highlights

• Pharmacological inhibition of Rac1 reduces AML cell migration to the hemopoietic niche.
• Rac1 inhibition and anthracycline combined acts synergistically to induce AML cell death.
• Rac1-inhibition combined with daunorubicin is superior to monotherapy *in vivo*.
• Rac1 inhibition could be a strategy to overcome therapy failure in AML treatment.

## Introduction

Acute myeloid leukemia (AML) is a blood- and bone marrow cancer characterized by the clonal expansion of immature myeloid cells. The standard chemotherapy regimen of seven days of cytarabine and three days of daunorubicin (DNR) was developed more than 50 years ago and has since then undergone only minor changes [22]. About half of AML patients still relapse within five years of diagnosis [2]. Relapsed AML and high age are associated with poor prognosis, where long-lasting curative outcome is an exception. In the last decade, molecular therapies targeting specific aberrant proteins in AML cells like FLT3, BCL-2, and IDH1/2 have been developed and implemented as additions to traditional chemotherapy or hypomethylating agents. However, some patients fail to respond to these therapies, for instance, because their leukemic blasts harbor no druggable target proteins or develop resistance after treatment [12,37].

The GTPase Ras-related C3 botulinum toxin substrate 1 (Rac1) is identified as a possible novel target for AML therapy [12]. This protein is commonly overexpressed in AML by upstream activation, such as from growth factors or via constitutively active mutated FLT3 [32]. Commonly, Rac1 is involved in the homing and engraftment of hematopoietic cells to the bone marrow niche downstream of the activated membrane receptors CXCR4 and CD44 [6,31]. Accordingly, Wang et al. demonstrated reduced homing to and interaction with the bone marrow niche in the AML cell line KG1-a with an induced dominant negative variant of Rac1, and furthermore, a reduced fraction of cells in a quiescent state. It has been shown that the transition of cells from a quiescent state to an active state renders them more vulnerable to chemotherapy [33]. Wu and colleagues found improved efficacy of anthracyclines when Rac1 was silenced with shRNA in a mouse model of AML [35]. Garitano-Trojaola et al. showed that overexpression of Rac1 altered the cytoskeletal organization of AML cells, the adhesion to surrounding cells, and induced the expression of the anti-apoptotic protein BCL-2. Combined FLT3-, BCL-2-, and Rac1 inhibition could re-establish drug sensitivity in a midostaurin (FLT3 inhibitor)-resistant MOLM-13 AML cell line [12].

Transient inhibition of Rac1 with the first-generation inhibitor NSC23766 was investigated by Cancelas et al. using a single intraperitoneal dose in a non-cancer C57BL/6 mouse model. This induced a doubling of hematopoietic stem cells and progenitors in the peripheral blood after six hours, returning to baseline values 24 h after treatment [7]. The second-generation Rac1 inhibitor EHop-016 is more potent and has demonstrated antiproliferative and proapoptotic effects in the low micromolar range on patient-derived AML cells [16]. It was reported as a synthesized derivate from NSC23766 in 2012. Also, it reduced directed cell migration in a breast cancer cell line at a concentration that did not affect the cell viability [24]. EHop-016 is more selective towards Rac1 than Eht1864, used by Garitano-Trojaola and colleagues, to study the migration of cancer cells [27]. Having established that Rac1 is an interesting target in AML, we wanted to investigate if Rac1 inhibition with EHop-016 could affect cell migration *in vitro* and the homing of AML cells to the hematopoietic niche *in vivo*. Furthermore, we wanted to investigate if combining Rac1-inhibition with DNR could give a synergistic anti-AML response.

Our investigations of leukemic cell migration were undertaken on the human AML cell line MOLM-13, which harbors an *FLT3*-ITD mutation, and using zebrafish larvae as a model system. The zebrafish (Danio rerio) has emerged as a useful and relevant tool for drug development and toxicity studies in cancer research. Compared to mammalian models, zebrafish have high fecundity, low cost, and easy maintenance, and their small size and transparency during the embryo and larval stage allow for *in vivo* single-cell studies using confocal microscopy [11]. In the early stages of development, from two days post fertilization (dpf) to five dpf, the caudal hematopoietic tissue (CHT) located in the ventral tail region is the main site of hematopoiesis. This is then gradually replaced by the thymus and caudal parts of the kidneys from three and four dpf, respectively [14]. Zebrafish have genetics compared to humans, with approximately 70 % of human genes having at least one orthologue in zebrafish [9]. Important for our research, Tulotta et al. have demonstrated cross-communication between human CXCR4 and zebrafish CXCL12 (ligand of CXCR4) in zebrafish xenografts [30].

## Method and materials

### Materials

The MOLM-13 cell line (DSMZ.no ACC 554, [23]) was obtained from the German Collection of Microorganisms and Cell Cultures GmbH (Braunschweig, Germany). After acquisition of the cells, they were authenticated by short tandem repeat analysis by the ATCC Cell Line Authentication Services and frozen in aliquots. The cells used in experiments were defrosted from these verified batches. Tricaine (cat.no: E10521), RPMI 1640 (cat.no: R5886), penicillin-streptomycin (cat.no: P0781 and P4458), L-glutamine (cat.no: G7513), dimethyl sulfoxide (DMSO) (cat.no: D2650), and fetal bovine serum (FBS) (cat.no: F7524 and S181B-500) were purchased from Merck KgaA (Darmstadt, Germany) and VWR (Radnor, PA, USA). Human CXCL12 (cat.no: 300-28A) was purchased from PeproTech Inc (Cranbury, NJ, USA). Flat-bottomed 96- and 24-well plates were from VWR (Radnor, PA, USA), µ-Slide 18-well confocal chamber slides from Ibidi (Gräfelfing, Germany), and transwell inserts with 8 µm pore size from Corning (Glendale, AZ, USA). The ^3^H-thymidine incorporation proliferation assay (cat.no: NET027A) was purchased from PerkinElmer Inc (Waltham, MA, USA), and the Annexin V/Propidium Iodide (PI) assay (cat.no: 640928) from Nordic BioSite (Täby, Sweden). For cell injections, micropipettes with a 13 µM inner diameter (cat.no: VESbv-13-0-0-55) were purchased from BioMedical Instruments (Zöllnitz, Germany). The cell staining kit CellTracker™ Deep Red Dye (cat.no C34565) was purchased from ThermoFisher Scientific (MA, USA).

EHop-016 (N4-(9-Ethyl-9H-carbazol-3-yl)-N2-(3-morpholin-4-yl propyl)-pyrimidine-2,4-diamine) was purchased from Merck KGaA (Darmstadt, Germany) and dissolved in DMSO to a stock concentration of 20 mM (8.6 mg/ml). DNR (Cerubidin®) from Sanofi (Paris, France) was dissolved in milli-Q water from Merck KGaA (Darmstadt, Germany) to a concentration of 8.87 mM (5 mg/ml). Stock solutions of the drugs were stored at -80 °C. The compounds were defrosted and diluted in RPMI 1640, phosphate-buffered saline (PBS), or E3 zebrafish medium on the day of the experiments.

### Cell maintenance and experimental conditions

#### Cell maintenance

The human AML MOLM-13 cell line was cultured at 37 °C with 5 % CO_2_ in RPMI 1640 medium supplemented with 10 % FBS, 50 IU/ml penicillin, 50 µg/ml streptomycin, and 0.2 mM L-glutamine. Routine testing for Mycoplasma infection was performed using a MycoAlert™ mycoplasma detection kit (cat.no: LT07-418, Lonza, Basel, Switzerland). No infection was detected during this study. The culture conditions during experiments were identical unless otherwise specified.

#### Proliferation assay

The ^3^H-thymidine proliferation assay was carried out in a flat-bottomed 96-well plate with 20 000 MOLM-13 cells per well. Vehicle control RPMI 1640, EHop-016, and/or DNR were added to a final 200 µl cell suspension volume in RPMI 1640 with 10 % FBS. The plates were incubated for 48 h before adding 20 µl (1 µCi) ^3^H-thymidine per well. After six h incubation, the plates were harvested, and nuclear incorporation was determined by liquid scintillation counting. Each experiment was performed in triplicate, and relative proliferation compared to untreated controls was calculated from the medians [28].

#### Flow cytometric detection of apoptotic cells

The Annexin V/PI assay was conducted to detect and quantify apoptosis. MOLM-13 cells suspended in culture media were seeded in flat-bottomed 24-well plates at 5.0 × 10^5^ cells/ml and added with vehicle control (RPMI 1640) or increasing concentrations of either EHop-016, DNR or a combination of both. The cells were incubated for 48 h before being harvested, placed on ice, and washed twice with 4 °C PBS. The cells were double stained with Annexin V and PI according to the manufacturer's protocol before data acquisition on a FACSVerse™ flow cytometer (BD Biosciences, San Jose, CA, USA). A total of 20 000 events were collected per sample. Flow cytometry data were handled in FlowJo v10.8.1 (BD, Ashland, OR, USA); data analysis included gating to remove debris and doublets and adjusting for autofluorescence of DNR using the AutoSpill function.

#### Measurement of MOLM-13 cell migration

MOLM-13 cells were seeded in a flat-bottomed 24-well plate at 1.0 × 10^6^ cells/ml. Vehicle control RPMI 1640 or EHop-016 was added to a final concentration of 6 µM or 8 µM in RPMI 1640 medium supplemented with 0.5 % FBS. The plate was incubated for 20 h. The cells were then harvested, centrifuged at 200 rcf for 5 min, and resuspended in RPMI 1640 without FBS. The viability was determined by counting the fraction of cells excluding trypan blue using a hemacytometer. The migration assay was performed in 24-well plates fitted with transwell inserts with 8 µm pore size. The upper compartment was loaded with 2.0 × 10^5^ viable cells in 200 µl RPMI 1640 without FBS. The lower compartment contained 600 µl of RPMI 1640 with either 10 % FBS or 100 ng/ml CXCL12. After six h of incubation, the cells in the lower well were collected by centrifugation, resuspended in 100 µl 0.9 % NaCl and counted with trypan blue exclusion. To ensure that the reduced cell count in the acceptor well was not due to cell death from the treatments, the cells were resuspended in RPMI with 10 % FBS without EHop-016 and cultured for 48 h before a new assessment of viability.

### Zebrafish larva handling and experimental conditions

#### Zebrafish larva handling

The zebrafish (*Danio rerio*) line Casper was used in this project [34]. Adult zebrafish were kept at, and fertilized zebrafish eggs obtained from, the zebrafish facility at the Department of Bioscience, University of Bergen. This facility is run according to the European Convention for the Protection of Vertebrate Animals used for Experimental and Other Scientific Purposes. The experiments on larvae were terminated at five days post fertilization (dpf), and thus did not require approval from the Norwegian Food Safety Authorities. Fertilized zebrafish eggs were incubated at 28.5 °C in E3 medium (5 mM NaCl, 0.17 mM KCl, 0.33 mM MgSO_4_, and 10 µM methyl blue as antifungal agent). Debris and dead larvae were removed daily. Between 24 h and 48 h post-fertilization, zebrafish larvae were dechorionated using forceps. During experiments with transplanted human cancer cells, the incubation temperature was raised to 31 °C. The zebrafish larvae were euthanized when reaching five dpf by cooling on ice for at least 30 min before being frozen at -20 °C overnight.

While the developing zebrafish is termed embryo prior to the protruding-mouth stage at 72 h post fertilization [20], for simplicity and to avoid confusion, zebrafish in all stages are called larva in this publication.

#### Transplantation of MOLM-13 cells by intravenous injection

Prior to injection, MOLM-13 cells were stained with CellTracker™ Deep Red according to the manufacturer's protocol. In brief, cells were centrifuged at 130 rcf for 5 min and resuspended in RPMI 1640 medium without FBS. The cell tracker staining solution was added to a final concentration of 20 µM, and the cells were incubated at 37 °C in a 5 % CO_2_ atmosphere for 30 min. Next, the cells were centrifuged and resuspended in RPMI 1640 medium with 10 % FBS. Cell suspensions of 1.0 × 10^7^ cells/ml in RPMI 1640 medium were injected using glass micropipettes with a beveled tip and inner diameter of 13 µm. The micropipettes were mounted in an injection setup with an MMO-220A micromanipulator system (Narashige, Tokyo, Japan), Eppendorf FemtoJet 4x microinjector (Eppendorf, Hamburg, Germany), and Leica M205 stereo microscope (Leica, Wetzlar, Germany). Injection pressure and time were adjusted to result in an injection droplet with a 200 µm diameter (approximately 4 nl) in peanut oil. Dechorionated zebrafish larvae were anesthetized using an E3 medium containing 0.7 mM tricaine before injection. The anesthetized zebrafish larvae were placed on 2 % agarose beds and injected into the posterior cardinal vein (PCV) (Fig. 3A, red arrow). Micropipettes for drug and PBS injections were self-drawn using a P-1000 micropipette Puller from Sutter Instrument (Novato, CA, US).

#### Toxicity assay of EHop-016 in zebrafish larvae

Two administration routes were tested to establish toxicity: intravenous injection and the addition of drugs in the E3 medium. For injections, zebrafish larvae at two dpf in the long pec stage [20] were injected intravenously with EHop-016 dilutions (10 µM or 100 µM in Milli-Q water) or Milli-Q water following the injection method described earlier in the method section. For in-water exposure, a dilution series of EHop-016 in E3 medium was created in a 96-well plate. One dechorionated zebrafish larva in the long pec stage was placed in each well. Visual toxic effects were assessed daily using microscopy, with abnormalities in the cardiovascular system (such as pericardial oedema, affections in heart-rate blood flow) and other visual abnormalities (such as developmental defects) being investigated.

To visualize blood flow, the larvae were sedated, and videos recorded under a microscope. The image series was filtered using a 3 × 3 median filter to reduce noise and new images were generated by calculating the standard deviation in pixel intensity across each time frame. Areas with high standard deviation indicate movement in the image along time frames. The standard deviation image was then overlayed on a still frame of the original image series and colored red. Image processing was performed in FIJI v. 2.14.0 [26].

#### Cell migration timelapse

The biodistribution of MOLM-13 cells in zebrafish larvae was investigated using a 12-hour timelapse. Dechorionated zebrafish larvae at one dpf in the pharyngula period [20] were incubated overnight in E3 medium with or without 20 µM EHop-016 in-water. The following day, CellTracker™ Deep Red stained MOLM-13 cells were injected into the PCV. Immediately following injection, the zebrafish larvae were placed in confocal chamber slides containing E3 medium with or without 20 µM EHop-016. Imaging was performed using an Andor Dragonfly 505 confocal system (Andor Technology, Belfast, Northern Ireland) equipped with an inverted Nikon Ti-E microscope with a Nikon CFI Plan Apochromat lambda 10x objective (Nikon, Tokyo, Japan). Imaging of the fluorescent cancer cells was performed using a 637 nm excitation laser and 700/38 nm band-pass filter. The timelapse was performed over 12 h, with image acquisition every 10 min. The number of MOLM-13 cells in the CHT was counted in a 724 µm x 121 µm (600 pix x 100 pix) bounding box positioned at the posterior end of the yolk sac and aligned with the ventral side of the notochord as shown in Fig. 3A (red box) at 0 h, three h, six h, and 12 h following the start of the timelapse.

#### *In vivo* EHop-016 efficacy assay

Zebrafish larvae were dechorionated at one dpf and the EHop-016 group pre-incubated overnight with 20 µM EHop-016 in E3 medium. The same day, MOLM-13 cells were stained with CellTracker™ Deep Red and further incubated in RPMI 1640 medium with 10 % FBS with or without 5 µM EHop-016. The following day, the prepared cells were injected into the zebrafish larva PCV. The EHop-016 group and combination group received cells pre-treated with EHop-016. The larvae were then imaged by confocal microscopy and injected with 4 nl 1 mM DNR or PBS into the PCV.

The zebrafish larvae were imaged using the same setup used for time-lapse imaging. The obtained confocal images were processed using an ImageJ plugin for batch-processing of cell segmentation [11], followed by statistical analysis.

#### Data analysis

Statistical analyses were performed in GraphPad Prism v. 9.5.1 (GraphPad Software, San Diego, CA, USA) and SPSS v. 27 (IBM Corp. Armonk, NY, USA). The half-maximal effective concentration (EC_50_) values were calculated from dose-response experiments using non-linear regression analysis. A two-way ANOVA with Tukey post hoc test was used to compare treatment groups from the Annexin V/PI assay, and with Fisher's Least Significant Difference (LSD) test to compare treatment groups for the *in vitro* cell migration data and the *in vivo* experiments. Paired *t*-tests were used to compare paired data before and after treatment. *P*-values < 0.05 were considered statistically significant.

The efficacy of the drugs and their combinations by the Annexin V/PI assay was determined by studying the combined percentages of apoptotic and necrotic cells in each sample, termed inhibition. Following analysis, the results for each sample were corrected with a weighted baseline correction calculated with the following formula:

$$I_{corrected}=\;I_{measured}-I_{baseline}*\frac{100-I_{measured}}{100-I_{baseline}}$$

Where $I_{corrected}$ is the inhibition after baseline correction, $I_{measured}$ the measured inhibition, and $I_{baseline}$ the average of the untreated controls.

The synergy score for cell experiments was determined by subtracting a calculated inhibition from the measured inhibition for each combination. The Bliss method was used to calculate a theoretical inhibition for each combination [3]. These calculations were performed using the web resource SynergyFinder Plus [38]. A score between -10 and 10 % indicates an additive effect, below −10 % an antagonistic effect, and above 10 % a synergistic effect.

## Results

### Effects of EHop-016 on MOLM-13 proliferation, viability and migration *in vitro*

**Fig. 1:**
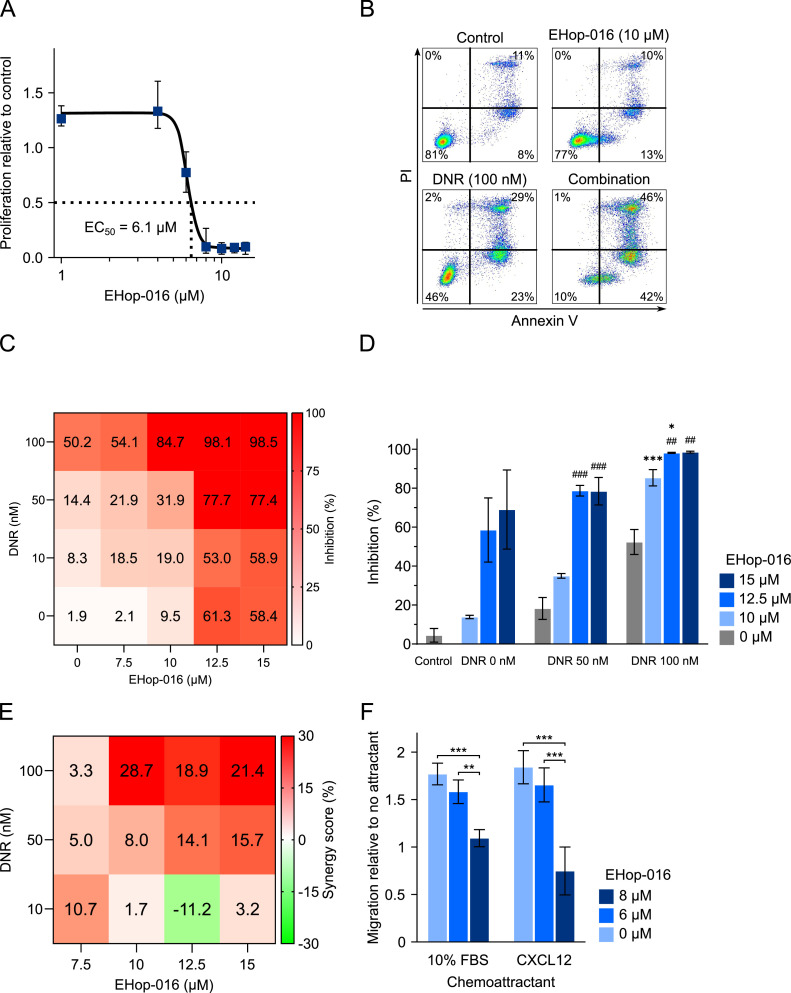
EHop-016 induces cell death and attenuates migration of MOLM-13 cells i*n vitro*. A: The antiproliferative effect of EHop-016 on MOLM-13 cells after 48 h incubation was measured using a ^3^H-thymidine assay. The data represented are relative to the untreated control and are the median of three replicates with a 95 % confidence interval. B–E: The reduction of viability and the induction of apoptosis and necrosis were evaluated using flow cytometric analyses on Annexin V/Propidium iodide (PI) stained cells. Before analysis, MOLM-13 cells were incubated for 48 h with EHop-016, Daunorubicin (DNR), or a combination. Gating to separate Annexin V and PI negative and positive cells was performed as shown in B. A heatmap of the percentage of non-viable cells (inhibition) according to different combination treatments is presented in C, each performed in duplicate. D: Selected combinations from C are presented as the mean and standard deviation. ^#^ indicates significant change compared with the same concentration of DNR alone, and * compared with the same concentration of EHop-016 alone, after a two-way ANOVA with the Tukey post hoc test. E: The synergy score was calculated as the deviation from the theoretical inhibition calculated by the Bliss method. Here, a synergy score of ±10 % indicates an additive interaction, below -10 % an antagonistic interaction, and above 10 % synergy. F: The ability of EHop-016 to inhibit migration was evaluated using a transwell migration assay. The MOLM-13 cells were incubated for 20 h with a deficit of FBS (0.5 %) and with or without EHop-016. Following incubation, the cells were left for six h to migrate to a lower well containing 10 % FBS or 100 ng/ml CXCL12 as chemoattractants or cell medium without FBS as a negative control. The ratio of migrated cells relative to the control is presented as the mean and standard deviation (*n* = 3). Significant change was calculated with a two-way ANOVA with Fisher's Least Significant Difference (LSD) test. *^/#^*p* ≤ 0.05, **^/##^*p* ≤ 0.01, ***^/###^*p* ≤ 0.001.

The ^3^H-thymidine assay demonstrated that EHop-016 had an antiproliferative effect on MOLM-13 cells after 48 h incubation with an EC_50_ of 6.1 µM (95 % confidence interval (CI) 5.9–6.4 µM) (Fig. 1A). To identify whether the reduced ^3^H-thymidine incorporation could be due to cell death, we investigated the viability and amount of apoptosis in cells treated with EHop-016 using the Annexin V/PI assay (Fig. 1B–E). EHop-016 reduced the number of viable MOLM-13 cells with an EC_50_ of 11.3 µM after 48 h incubation (data not shown), with gating to separate viable and non-viable cells as shown in Fig. 1B. Next, MOLM-13 cells were treated with EHop-016 in combination with DNR for 48 h to reveal synergistic effects. The baseline corrected results of the apoptosis assay are shown in Fig. 1C and are the average of two replicates for each combination. A two-way ANOVA with Tukey post hoc test to correct for multiple testing revealed a significant change in the number of non-viable (inhibited) cells when adding EHop-016 12.5 and 15 µM to DNR 50 or 100 nM, as compared with the same concentrations of DNR alone (*p*-values < 0.01). Likewise, adding DNR 100 nM to EHop-016 10 or 12.5 µM significantly increased the number of inhibited cells compared to EHop-016 alone (*p*-values < 0.05) (Fig. 1D). The Bliss synergy score in Fig. 1F was calculated using the baseline corrected values in 1C. For the combinations including 7.5 µM EHop-016 or 10 nM DNR a synergy score within the range of ±10 % was observed, indicating an additive interaction at these concentrations. At higher concentrations of both drugs, except for the 50 nM DNR and 10 µM EHop-016 combination, the score was higher than 10 % indicating a synergistic interaction.

Since Rac1 is involved in hematopoietic cell migration, we next investigated to which extent EHop-016 affected the migration of MOLM-13 cells using FBS or CXCL12 as chemoattractants. Untreated cells preferred 10 % FBS and CXCL12 to RPMI without attractants (fold change > 1.5). Pretreatment for 20 h with 8 µM EHop-016 reduced the migration toward both chemoattractants compared with their untreated controls (*p* < 0.001 towards 10 % FSB and *p* < 0.001 towards CXCL12, two-way ANOVA with Fisher's LSD test) and compared with 6 µM treatment (*p* = 0.003 towards 10 % FBS and *p* < 0.001 towards CXCL12) (Fig. 1F). To assess the viability of the treated cells, they were washed and resuspended in RPMI with 10 % FBS without EHop-016 for 48 h. The fraction of viable cells was not affected by EHop-016 compared to untreated control cells (data not shown).

### Toxic effects of EHop-016 on zebrafish larvae

**Fig. 2:**
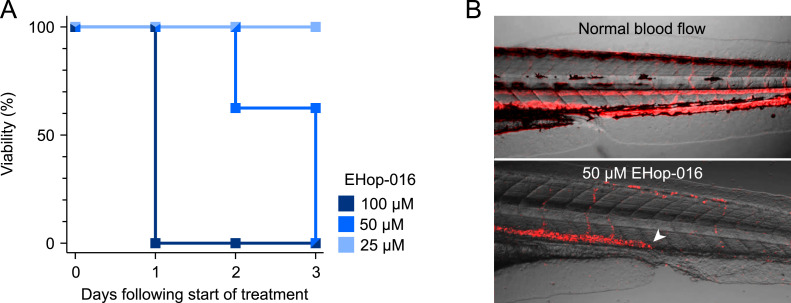
Toxicity of EHop-016 in zebrafish larvae. Zebrafish larvae were dechorionated at two dpf and placed in a 96-well plate with one larva per well. EHop-016 diluted to different concentrations in E3 medium was added to each well. The zebrafish larvae were screened daily for visual toxic effects until euthanasia at five dpf. A: Viability of the zebrafish larvae incubated in different concentrations of EHop-016 for three days. n = 8 larvae in each group. B: Visualization of normal and obstructed blood flow from video recording of larvae without treatment or treated with 50 µM EHop-016 for 24 h. Visualization was done by overlaying the standard deviation in pixel intensity across time frames (red) on a still video frame. Normal blood flow is shown above, while a larva treated with 50 µM EHop-016 is shown below. The white arrow marks the point of obstructed blood flow in the caudal hematopoietic tissue (CHT).

Before commencing the efficacy studies on zebrafish larvae, we needed to establish the maximum tolerable dose (MTD) of EHop-016 in zebrafish larvae. The toxic effects were investigated for both in-water and intravenous administration. In-water administration started at two dpf. Administration of 100 µM EHop-016 in E3 medium resulted in the death of all larvae during the first 24 h of exposure (Fig. 2A). Of the eight larvae treated with 50 µM, three were found dead two days after administration, and the remaining died three days after drug administration. In the larvae surviving 50 µM treatment until two days after administration, we observed adverse effects on the circulatory system. One of the surviving larvae had a cardiac arrest and subsequently no blood circulation. Additionally, two other larvae had heartbeats but no blood circulation, and an obstruction was detected in the ventral tail area (Fig. 2B and Supplementary Videos S1 and S2). None of the larvae exposed to EHop-016 concentrations below 50 µM died or showed signs of toxicity like circulatory defects during the experiment. Additionally, zebrafish larvae were injected intravenously with 4 nl 100 µM EHop-016, 10 µM EHop-016, or Milli-Q water at two dpf. No toxic effects were observed in these larvae until euthanasia at five dpf. Based on these results, we determined the MTD of EHop-016 in zebrafish larvae to be 25 µM when administered in-water and at least 100 µM as one 4 nl bolus intravenous injection.

### EHop-016 reduces the accumulation of MOLM-13 cells in the caudal hematopoietic tissue

**Fig. 3:**
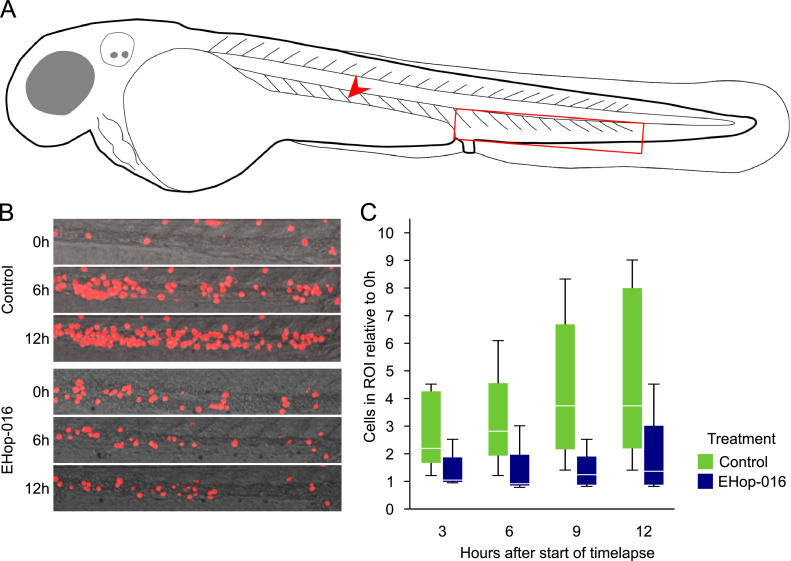
Inhibited migration of injected MOLM-13 cells to the CHT. Zebrafish larvae were dechorionated and incubated in E3 medium with or without 20 µM EHop-016. The following day, the zebrafish larvae were injected with MOLM-13 cells stained using CellTracker™ Deep Red. Immediately following cell transplantation, a 12-hour timelapse was conducted with images of the tail regions acquired every 10 min using confocal microscopy. The number of transplanted cancer cells was determined in a 724 µm x 121 µm (600 pix x 100 pix) region in the tail including the CHT. This region of interest (ROI) is illustrated as a red rectangle in A, and a red arrow indicates the injection site. In B: confocal images show accumulation of MOLM-13 cells in the caudal hematopoietic tissue (CHT) of a control and an EHop-016-treated larva at zero, six, and 12 h after injection. **C**: For each zebrafish larva, the count of MOLM-13 cells in the ROI was calculated relative to the count at the start of the timelapse. The data are shown as boxplots, *n* = 5. See the main text for description of statistical significance.

To investigate how EHop-016 treatment affected cell migration *in vivo*, a 12-hour timelapse was recorded on zebrafish transplanted with fluorescently labeled MOLM-13 cells. We have previously shown that MOLM-13 cells accumulate to the CHT (see Fig. 3A for localization in the larvae) after intravenous injection [11], where hematopoiesis occurs in the zebrafish larvae. We found that MOLM-13 cells accumulated in the CHT during the first 12 h after transplant (Fig. 3B and C). However, in larvae treated with 20 µM EHop-016 in-water, there was less accumulation of cells in the CHT. A comparison of the number of cells in the CHT of EHop-016-treated larvae at 12 h and the start of the experiment gave no significance (paired *t*-test). Untreated larvae had a significantly higher number of cells in the CHT than EHop-016 treated larvae throughout the experiment (*p* < 0.01, two-way ANOVA).

### The combination of EHop-016 and DNR reduces the tumor burden *in vivo*

**Fig. 4:**
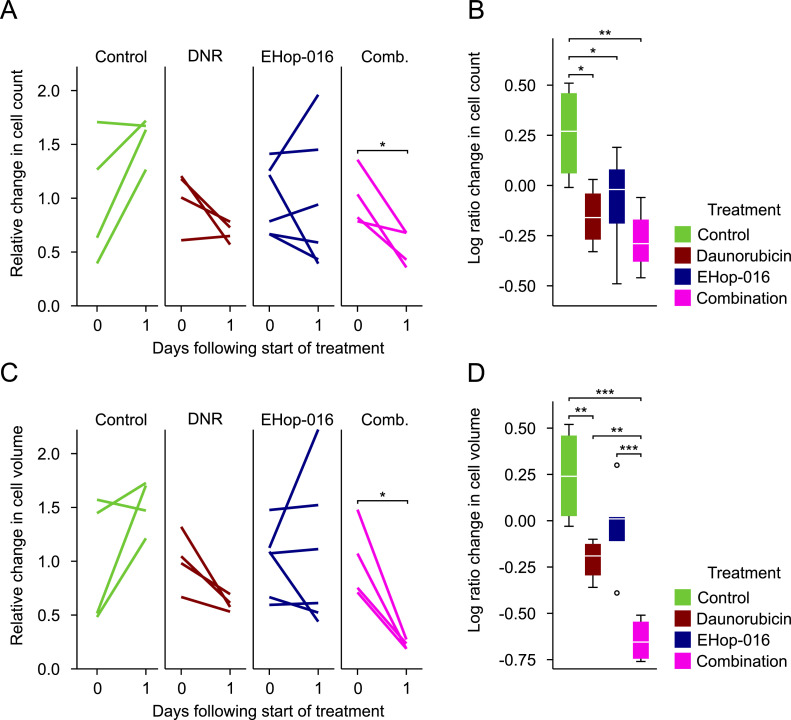
*In vivo* efficacy of EHop-016 and DNR alone or in combination. Zebrafish larvae at one dpf were dechorionated and divided into four groups: Control without treatment, single treatment with EHop-016 or daunorubicin (DNR), and a combined treatment of both drugs. Larvae in the EHop-016 and combination group were pre-treated with 20 µM EHop-016 in E3 medium for 24 h before injection with MOLM-13 cells stained with CellTracker™ Deep Red. The cells in the EHop-016 and combination group were also pre-treated with 5 µM EHop-016. At two dpf, the fluorescently stained cells were intravenously injected into the zebrafish larvae. Larvae in the DNR and combination group were injected with 4 nl 1 mM DNR in PBS. Images were acquired at two dpf and three dpf using confocal microscopy. The resulting images were processed as described in the methods section and in [11]. Larvae that had ten or fewer cancer cells immediately after injection were excluded. The change in cancer cell count and total cancer cell volume are shown in A and C, respectively, with larvae in each group represented by a unique color. All values were taken relative to the respective average for all larvae in the group at two dpf. A log-transform of the relative changes in each group for cancer cell count and volume are shown in boxplots B and D, respectively. n = 4 for all groups except EHop-016, where *n* = 6. For statistical analysis, a paired samples test was performed for the line plots in A and C, and an ANOVA with Fisher's LSD test in B and D. * *p* ≤ 0.05, ** *p* ≤ 0.01, *** *p* ≤ 0.001. If no significance is indicated, *p* > 0.05, *i.e.* not significant.

The proapoptotic effect of EHop-016 by itself and in combination with DNR was evaluated in zebrafish larvae injected with MOLM-13 cells. To ensure optimal drug exposure to EHop-016, we utilized pretreatment of both larvae and cancer cells. Progression of tumor burden can be represented as AML cell count or total volume of AML cells, both relative to the count of AML cells before treatment. The two approaches were used since the segmentation process can in some cases lead to nearby cells being counted as one, thereby influencing the cell count, while cell volume measure can sometimes be affected by changes in fluorescence intensity. The relative change of cell count or total volume of MOLM-13 cells (Fig. 4A and C, respectively) was calculated to find the tumor progression over the first 24 h. Of the four treatments (vehicle control, DNR, EHop-016, and combination of DNR and EHop-016), only the combination of DNR and EHop-016 significantly reduced the tumor burden (*p* = 0.043 for cell count and 0.018 for cell volume, paired sample *t*-test, see Fig. 4A and C, respectively). No significant changes in tumor burden were found in control larvae or larvae treated with EHop-016 (20 µM in-water) alone. There was an apparent decrease in tumor burden in larvae treated with DNR without EHop-016 (4 nl 1 mM injection of DNR), but this was not significant when compared with tumor burden before treatment (paired sample *t*-test: *p* = 0.12 for count and 0.054 for volume).

To compare how the different treatments affected tumor burden, the relative changes in MOLM-13 cell count and total volume from two dpf to three dpf were log-transformed. Compared to the control group, the groups receiving treatment, except for volume measurements of EHop-016 alone, exhibited a significantly lower AML cell count and total volume (Fig. 4B and D). Furthermore, the larvae treated with EHop-016 and DNR in combination had a significantly lower total cell volume than those treated with either drug alone (*p* < 0.001 compared with EHop-016 and *p* = 0.007 compared with DNR) (Fig. 4D). Taken together, these data show that combining DNR with EHop-016 is more efficient than either drug alone.

## Discussion

In this study, we demonstrate the potential of pharmacological inhibition of Rac1 in AML therapy using the molecule EHop-016 on MOLM-13 AML cells *in vitro* and *in vivo*. The zebrafish larva was chosen as a model system for the *in vivo* studies, particularly since they allow for high-resolution visualization of single-cell migration. EHop-016 was administered to the larvae by adding it to the water. The observed toxic effects of in-water treatment indicate uptake of the drug. Even though it is difficult to precisely determine the concentration in the zebrafish larval circulation after in-water administration, it will reduce the unnecessary stress of repeated drug injections and ensure continuous exposure to the drug. This amends for the short half-life of EHop-016 found in mice, about 4.5 h after intraperitoneal administration, presumably due to combined renal and hepatic elimination [17]​.

In the zebrafish larvae, the CHT is the major site of hematopoiesis between two to five dpf, thereby serving as a likely migration target for leukemic cancer cells [14], including MOLM-13 [11]​. Our finding that EHop-016 inhibits AML cell migration *in vitro* (Fig. 1F) was also confirmed *in vivo* (Fig. 3). However, whether the observed reduction of accumulation in the CHT was due to altered attraction or anchoring to the CHT, altered migration of the cells, or modulated cell-to-cell adhesion in the vascular endothelium is not known ​ [29]. While zebrafish have endothelial and stromal cells producing chemoattractants like CXCL12, this production is low in the early larval stage [13], which could suggest that our observations are due to generally reduced cell motility rather than reduced response to chemoattractants.

The antiproliferative and proapoptotic effects of EHop-016 *in vitro* are in line with reported findings on other cancer cells. EHop-016 decreased the cell viability and induced apoptosis on breast cancer cell lines [4] and gallbladder cancer cell lines [19]. Likewise, EHop-016 reduced the tumor burden in a breast cancer mouse model [8]​. Our study demonstrates that EHop-016 potentiates the effect of DNR (Fig. 1D and E) and induces a significantly decreased tumor burden *in vivo* in the combination treatment group (Fig. 4). Combination treatment was previously successful with EHop-016 and paclitaxel in a lung cancer cell line [36], with NSC23766 and cisplatin and carboplatin in breast and lung cancer cell lines [21] and with Eht1864 and DNR in the AML cell line OCI-AML3 [25]. No *in vivo* experiments with the combination of Rac1 inhibition and anthracyclines have previously been reported. Still, our data suggests that this may be a promising therapeutic strategy, which merits investigations in other AML cell lines and preferably a panel of AML patient blasts to strengthen the findings. It is not unlikely that the combination of anthracyclines and EHop-016 could be effective in other cancers as well.

The concept of inhibiting leukemic cell attachment to the bone marrow niche combined with chemotherapy has been investigated using other targets and drugs. In AML, one of the obstacles for successful treatment is insufficient eradication of the quiescent leukemic stem cells (LSC) from the bone marrow niche or extramedullary sites, where they evade the toxic effects of chemotherapy, leading to a later relapse or refractory disease [33]. The CXCR4 antagonist BL-8040 is currently being investigated with cytarabine in clinical trials for AML to induce mobilization of leukemic blasts into the peripheral blood [5]​. DeAngelo and colleagues have reported the encouraging clinical results of an inhibitor of the cell adhesion molecule E-selectin/CD62E, also present in leukemic cells and LSCs ​ [10].​ However, the advantage of targeting a downstream effector molecule, like Rac1, is that it affects events downstream of several adhesion molecules and receptors. Thus, inhibition might both reduce the homing to the hematopoietic niche and induce a more vigorous mobilization of leukemic cells and the LSC, as seen by Jia and colleagues when they combined targeting of CXCR4 and E-selectin/CD62E *in vitro* in AML ​ [18].

At an in-water concentration of 50 µM, toxic effects occurred in the zebrafish larvae, manifested as reduced and sometimes blocked blood flow (Fig. 2B). This might relate to the decreased endothelial tube formation, which has been demonstrated on umbilical vein endothelial cells after 24 h treatment with EHop-016 ​ [8]​. This study also reported a reduction in the number of blood vessels on the surface of breast cancer tumors in a mouse model treated with EHop-016. While no toxicity was noted in the adult mice, our model's zebrafish larvae are still undergoing development with substantial angiogenesis, which could increase their susceptibility to this effect. We have no indications that the reduced blood circulation is due to aggregation of thrombocytes, present in zebrafish from 36 h post fertilization ​ [15]. On the contrary, the first-generation Rac1-inhibitor NSC23766 inhibited platelet aggregation and lamellipodia formation induced by thrombin ​ [1].

No Rac1 inhibitor has so far entered a clinical trial. The short half-life of EHop-016 in mice might suggest a twice-a-day dosing regimen, an infusion, or a controlled-release formulation to maintain sufficient time for the drug to exert the desired effect on AML cells. Our experiment was performed with DNR in combination with EHop-016, but possible longer-term effects with other drug combinations, such as both DNR and cytarabine or a BCL-2 inhibitor, could reveal even greater therapeutic potential.

## Conclusion

The signaling molecule Rac1 is an interesting target in AML, being implicated in several cellular processes and downstream of many receptors. The Rac1 inhibitor EHop-016 can attenuate the migration of MOLM-13 cells to the CHT in a zebrafish larva model and likely therefore enhance the proapoptotic effect of DNR both *in vitro* and *in vivo*. Our research supports the notion from other experiments that Rac1 targeting can contribute to improved therapy outcomes in AML and should be further evaluated by *in vivo* studies.

## Funding

This research received funding from Western Norway Regional Health Authority to ALH (Grant no. F-11001) and JLF (Grant no. F-12533), and from the Norwegian Society for Children's Cancer to LH (Grant Nos. 180007 and 190004). The funders had no role in study design, analysis or preparation of the manuscript.

## CRediT authorship contribution statement

**Anette Lodvir Hemsing:** Conceptualization, Formal analysis, Investigation, Methodology, Visualization, Writing – original draft, Writing – review & editing. **Jan-Lukas Førde:** Conceptualization, Formal analysis, Investigation, Methodology, Software, Visualization, Writing – original draft, Writing – review & editing. **Håkon Reikvam:** Conceptualization, Methodology, Resources, Supervision. **Lars Herfindal:** Conceptualization, Formal analysis, Methodology, Resources, Supervision, Writing – original draft, Writing – review & editing.

## Declaration of competing interest

The authors declare that they have no known competing financial interests or personal relationships that could have appeared to influence the work reported in this paper.

## Appendix Supplementary materials

Image, video 1
